# Advanced progress of the relationship between PCSK9 monoclonal antibodies and hyperglycemic adverse events

**DOI:** 10.3389/fcvm.2023.1117143

**Published:** 2023-06-26

**Authors:** Ruixing Zhang, Yongxiang Wang, Yu Peng, Jing Zhao, Zheng Zhang

**Affiliations:** ^1^ The First Clinical Medical College, Lanzhou University, Lanzhou, China; ^2^ Department of Heart Center, The First Hospital of Lanzhou University, Lanzhou, China; ^3^ Key Laboratory for Cardiovascular Diseases of Gansu Province, The First Hospital of Lanzhou University, Lanzhou, China

**Keywords:** proprotein convertase subtilisin/kexin type 9 monoclonal antibodies, lipid metabolism, hyperglycemic adverse events, coronary heart disease, type 2 diabetes mellitus

## Abstract

**Purpose of Review:**

Long-term use of statins had been confirmed to cause an increase in hyperglycemic adverse events (HAEs), whose mechanism has been well understood. Proprotein convertase subtilisin/kexin type 9 (PCSK9) monoclonal antibodies (PCSK9-mAbs), a kind of new lipid-lowering drug, can effectively reduce plasma low-density lipoprotein cholesterol levels in patients with CHD and have been widely used. However, animal experiments, Mendelian randomization studies, clinical researches and Meta-analyses which focused on the relationship between PCSK9-mAbs and HAEs had reached different conclusions, which has attracted great attention from clinicians.

**Recent Findings:**

The newest FOURIER-OLE randomized controlled trial followed PCSK9-mAbs users for over 8 years, whose results suggested that long-term use of PCSK9-mAbs did not increase the incidence of HAEs. Newest Meta-analyses also indicated that there was no relationship between PCSK9-mAbs and NOD. Meanwhile, genetic polymorphisms and variants related to PCSK9 might have effects on HAEs.

**Conclusion:**

According to the results of current studies, there is no significant relationship between PCSK9-mAbs and HAEs. However, longer-term follow-up studies are still needed to confirm it. Although PCSK9 genetic polymorphisms and variants may affect the possible occurrence of HAEs, there is no need to perform relevant genetic testing before applying PCSK9-mAbs.

## 1. Introduction

Dyslipidemia is an important cause of coronary heart disease (CHD). Therefore, lipid control is extremely important in the long-term management of CHD. Type 2 diabetes mellitus (T2DM), as one of the highest risk factors of CHD, is also a common complication in patients with it.

**Figure 1:**
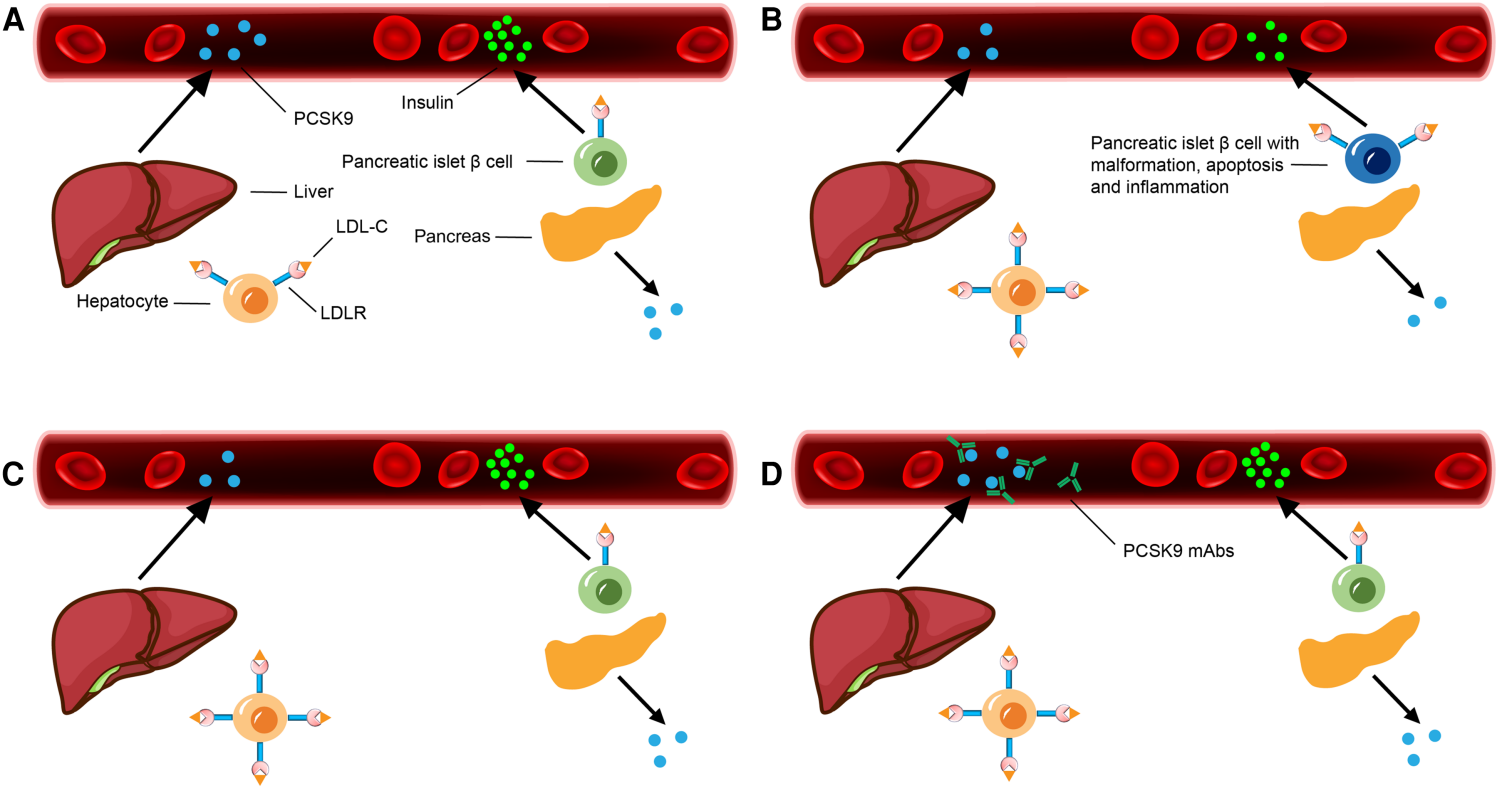
PCSK9 secretion of liver as well as pancreas and insulin secretion of pancreatic islet β cells in different situations. (**A**) Under normal physiological state. (**B**) Under physiological state of PCSK9^−/−^ gene individual: PCSK9 secretion of liver and pancreas decreases, LDLR abundance on the surface and LDL-C endocytosis of hepatocytes as well as pancreatic islet β cells increase, with insulin secretion of pancreatic islet β cells decreases significantly. (**C**) After PCSK9 LSKO: PCSK9 secretion of liver decreases, LDLR abundance on the surface and LDL-C endocytosis of hepatocytes increase. LDLR abundance on the surface, PCSK9 secretion and insulin secretion of pancreatic islet β cells are normal. (**D**) After applying PCSK9-mAbs: PCSK9 secretion of liver is normal. The secreted PCSK9 is bound with PCSK9-mAbs and degrades in circulation, with LDLR abundance on the surface and LDL-C endocytosis of hepatocytes increase. LDLR abundance on the surface, PCSK9 secretion and insulin secretion of pancreatic islet β cells are normal.

Statins have a clear and reliable effect on lowering plasma low-density lipoprotein cholesterol (LDL-C) levels, and have been used as the first-choice lipid-lowering drugs in clinical practice for more than 30 years. However, long-term use of statins has been confirmed to cause an increase in hyperglycemic adverse events (HAEs), including impaired fasting plasma glucose (FPG), increased glycated haemoglobin (HbA_1c_), and even new-onset diabetes (NOD), through various mechanisms such as inhibiting plasma glucose uptake, promoting insulin resistance, and inhibiting insulin secretion (1, 2). In human's circulation, after LDL-C in plasma is bound to LDL receptor (LDLR) on the surface of hepatocytes, it will be endocytosed into hepatocytes and hydrolyzed by lysosomal hydrolases (3). In 2003, Abifadel et al. discovered a human protein convertase playing an important role in lipid metabolism, which was named proprotein convertase subtilisin/kexin type 9 (PCSK9) (4). PCSK9 is mainly produced and secreted by hepatocytes, to increase plasma LDL-C levels by reducing LDLR abundance on the surface of hepatocytes (Figure 1A). PCSK9 monoclonal antibodies (PCSK9-mAbs) are one kind of PCSK9 inhibitors. As a new lipid-lowering drug, it can bind to plasma soluble PCSK9 molecules, preventing them binding to LDLR and reducing degradation of them. In this way, PCSK9-mAbs may increase intracellular transport of LDL-C and reduce concentration in plasma (5). Nowadays, there are kinds of PCSK9 inhibitors, including three PCSK9-mAbs, alirocumab, evolocumab and bococizumab, as well as PCSK9 small interfering RNA (siRNA), inclisiran, and so on. In large clinical trials, PCSK9-mAbs have shown 60% LDL-C reduction and 20%–25% decrease in major cardiovascular events (MACEs) (6–8). Among them, alirocumab and evolocumab had been approved by the US Food and Drug Administration as well as European Medicines Agency in 2015 for treating patients with familial hypercholesterolemia (FH), statin intolerance or contraindication, and atherosclerotic cardiovascular diseases.

However, animal experiments, Mendelian randomization studies, clinical researches and Meta-analyses which focused on the relationship between PCSK9-mAbs and HAEs had reached different conclusions, which has attracted great attention from clinicians. This article mainly reviews advanced progress of the relationship between PCSK9-mAbs and HAEs.

## 2. Research status of the relationship between PCSK9 & PCSK9-mAbs and HAEs

### 2.1. Animal experiment

Previous experiment had shown that, pancreatic islet β cells expressed significant amounts of LDLR (Figure 1A). LDLR could mediate uptake of exogenous lipoproteins through established pancreatic islet β cell lines as well as isolated and cultured islets. Long time exposure to high LDL-C or very low-density lipoprotein cholesterol (VLDL-C) level was lethal for these cells, and this effect was LDLR dependent. However, high density lipoprotein cholesterol (HDL-C) appeared to have a protective effect (9). In an animal experiment, a semi-quantitative immunoblotting (sqIB) analysis of proteins extracted from pancreatic tissue displayed that PCSK9^−/−^ mice expressed twofold more LDLR abundance in this organ than PCSK9^+/+^ mice. Besides, compared with PCSK9^+/+^ mice, in pancreas of PCSK9^−/−^ male mice older than 4 months, there was more LDLR and less insulin. In this way, PCSK9^−/−^ male mice were with hypoinsulinemia, hyperglycemia and glucose intolerance. At the same time, their pancreatic islets exhibited signs of malformation, apoptosis and inflammation under microscope (10). These results suggest that, systemic defects in PCSK9 may negatively affect function of pancreatic islet β cells, with harmful effects on glucose homeostasis (Figure 1B).

Another gene knockout (KO) animal experiment focused on glycemic changes in PCSK9 KO mice and albumin AlbCre+/PCSK9^LoxP/LoxP^ (PCSK9 liver selective KO, PCSK9 LSKO) mice. Compared with WT mice, after 20 weeks, PCSK9 KO mice given a standard or high-fat diet both showed significantly impaired glucose clearance, increased insulin levels in pancreatic islet β cells, and decreased insulin as well as C-peptide levels in circulation. As for PCSK9 LSKO mice, PCSK9 levels in circulation were below the limit of detection while maintaining PCSK9 production in other organs, including pancreas (Figure 1C). This situation is very similar to patients treated with PCSK9-mAbs, in which PCSK9 is absent from circulation but can still be produced in extrahepatic organs. Under this condition, their insulin levels in circulation and pancreatic islet β cells, LDLR abundance on the surface of pancreatic islet β cells and LDL-C levels in pancreatic islet β cells were similar to those of WT mice. Thus, glucose tolerance and insulin tolerance also remained normal (11). These results indicate that plasma PCSK9 affects, if any, LDLR abundance on the surface of pancreatic islet β cells slightly, and suggest that anti-PCSK9 therapies targeting circulating PCSK9 may affect LDLR abundance on the surface of pancreatic islet β cells limitedly. These also suggest that circulating and liver-derived PCSK9, the principal targets of PCSK9-mAbs, does not have a significant effect on function and insulin secretion of pancreatic islet β cells.

In this way, the key to determining whether plasma glucose is elevated is whether PCSK9 secretion of pancreatic islet β cells is absent, which directly affects LDLR abundance on the surface of pancreatic islet β cells and cholesterol metabolism balance. The commercial PCSK9-mAbs targeting hepatic PCSK9 deficiency don't affect PCSK9 secretion of pancreatic islet β cells, indicating that application of PCSK9-mAbs is safe.

### 2.2. Mendelian randomization study

A Meta-analysis showed that, exposure to LDL-C lowering genetic variants in or near NPC1L1 as well as other genes was related to higher risk of T2DM. Meanwhile, it found that, there was a relationship between PCSK9 variants and higher risk of T2DM. This research pointed out that, unlike the relationship between LDL-C lowering alleles and cardiovascular risk, the relationship between these alleles and metabolic risk appeared to be gene-specific, which might suggest there were target-specific adverse consequences of LDL-C lowering agents on the risk of T2DM (12).

Further Mendelian randomization study found that, PCSK9 variants and 3-hydroxy-3-methylglutaryl–coenzyme A reductase (HMGCR) variants had roughly the same effect on the risk of MACEs and T2DM per unit decrease in LDL-C level, whose scores had additive effects. Based on this, they made an assumption that PCSK9-mAbs might increase the risk of NOD like statins too. However, the increased risk of NOD associated with PCSK9 and HMGCR variants appeared to be limited in patients with impaired FPG. Thus, as with statins, any potential increase in the risk of NOD during PCSK9-mAbs therapy might be limited in patients with impaired FPG too. Meanwhile, this study also pointed out that, lifetime exposure to reduced LDL-C levels mediated by genetic variants was related to a much greater reduction in the risk of CHD per unit decrease in LDL-C level than short-term PCSK9-mAbs therapy. In this way, it gave an assumption that, effect of estimated PCSK9 variants on the risk of MACEs (and probably NOD) was likely to be quantitatively much larger than effect of PCSK9-mAbs treatment, measured by unit decrease in LDL-C level, which has been observed in ongoing outcome trials (13).

Another Mendelian randomization study, with a sample size of more than 600,000 patients, conjointly analyzed four independent PCSK9 variants (rs2479409, rs11206510, rs11583680, and rs11591147). This approach assumed additive effects for all single nucleotide polymorphisms, which were well validated in sensitivity analyses. This study concluded that, genetic variants in PCSK9 were related to lower LDL-C levels in plasma and higher risk of T2DM. And these variants were also related to higher FPG, bodyweight, as well as waist-to-hip ratio (14). However, just as another article presented, this study also pointed out that, effect estimates obtained from several Mendelian randomization studies could only represent lifetime exposure to natural genetic variants. Therefore, it might not be directly translated to effect size of any corresponding treatment introduced much later in life since its shorter duration (14, 15).

The newest Mendelian randomization study showed that, for PCSK9-mAbs, LDL-C lowering per standard deviation decrease in PCSK9 expression indicated a modest T2DM risk reduction. However, after adjustment for other lipids and physical activity, the relationship between PCSK9-mAbs application and T2DM was not observed (16). This suggests that the use of PCSK9-mAbs may not be associated with increased risk of HAEs.

### 2.3. Clinical research and Meta-analysis

As for alirocumab, an early randomized controlled trial, ODYSSEY, followed 3,448 patients without T2DM at baseline for 6 to 18 months. The results showed that, compared with placebo or ezetimibe, effect of alirocumab on transition to NOD couldn't be proved (17). And a prespecified analysis of ODYSSEY randomized controlled trial, including 18,924 patients with CHD, with or without T2DM at baseline, suggested that alirocumab reduced twice MACEs among patients with T2DM as in those without T2DM. At the same time, alirocumab did not increase the risk of NOD (18). Furthermore, a post-hoc subanalysis of ODYSSEY randomized controlled trial conducted in Japan revealed that, the use of alirocumab for 52 weeks resulted in reductions of apolipoprotein B, non-HDL-C, and lipoprotein(a) levels as well as increases of HDL-C and apolipoprotein A-1 levels in patients with or without T2DM at baseline. Importantly, this analysis did not find any apparent association between the use of alirocumab and HAEs (19). However, a further study based on ODYSSEY randomized controlled trial showed that, baseline lipoprotein(a) level might have effects on the relationship between alirocumab and HAEs: at low baseline lipoprotein(a) levels, alirocumab seemed to reduce incidence of T2DM; while at high baseline lipoprotein(a) levels, alirocumab seemed to increase incidence of T2DM (20). When it comes to evolocumab, FOURIER randomized controlled trial followed 27,564 patients for 2.2 years, concluding that it did not increase the risk of NOD in patients without T2DM at baseline, including those with prediabetes (21). The newest FOURIER-OLE randomized controlled trial showed that, compared with the placebo control group, using evolocumab over 8 years significantly reduced incidence of MACEs. At the same time, long-term use of evolocumab did not increase incidence of NOD, suggesting that it was not significantly related with HAEs (22).

When referring to whether the dose of PCSK9-mAbs have effects on HAEs, one clinical research based on ODYSSEY randomized controlled trial found that, compared with those who did not receive alirocumab, there was no significant changes in FPG and HbA_1c_ among patients applied alirocumab at the largest dose (300 mg every 4 weeks) (23). What's more, FOURIER-OLE randomized controlled trial also indicated that, using evolocumab over 8 years at normal dose (140 mg every 2 weeks or 420 mg every 4 weeks) would not increase the incidence of HAEs (22).

Recent comparative drug retrospective research showed that, in a real-world setting, HAEs were reported more common in PCSK9-mAbs treatment compared with ezetimibe, but less common compared with statins. When PCSK9-mAbs were subdivided into alirocumab and evolocumab, this research found that it was evolocumab, but not alirocumab, was associated with HAEs. Notably, HAEs were reported more frequently in patients with T2DM than in patients without T2DM, with most occurring within 6-month treatment and being reversible with drug discontinuation. Meanwhile, this study also emphasized that, PCSK9-mAbs therapy was related to increased report of mild hyperglycemia, but not NOD (24). Furthermore, one clinical study found that, after 1-year treatment, insulin resistance of patients using statins monotherapy was significantly increased, while insulin resistance in patients who applied ezetimibe and statins was no significantly increased, even tended to decrease (25).

Two earlier Meta-analyses both showed that alirocumab and evolocumab had no significant effect on incidence of T2DM and glucose metabolic disorder (26, 27). Another Meta-analysis suggested that, application of PCSK9-mAbs would lead to a small but significant increase in FPG and HbA_1c_ in short term, but this effect was not sufficient to increase incidence of NOD (28). The results of two newest Meta-analyses, including 30 and 32 randomized controlled trials respectively, also indicated that there was no relationship between PCSK9-mAbs and NOD (29, 30). What's more, one Meta-analysis took PCSK9-mAb type into consideration, which found that alirocumab was associated with a significant reduction in the risk of NOD, whereas no significant reduction was observed in evolocumab (31), suggesting that the type of PCSK9-mAbs might have effects on the relationship between PCSK9-mAbs and HAEs. The reason why this happened might be attributed to that the body produced different anti-drug antibodies after applying two different types of PCSK9-mAbs.

According to the current researches, it can conclude that, applying PCSK9-mAbs at permitted dose does not cause significant NOD or T2DM, with the worst effect being a mild increase in FPG.

### 2.4. Other PCSK9 inhibitors

Inclisiran, a PCSK9 siRNA, is a novel post-transcriptional gene silencing therapy, which has a powerful, dose-dependent, long-lasting effect in lowering LDL-C by inhibiting the synthesis of PCSK9 through RNA interference. Inclisiran is a chemically synthesized siRNA that is covalently linked to a ligand containing three N-acetylgalactosamine residues. These residues lead to hepatocyte specific uptake of siRNA, where inclisiran binds to the RNA-induced silencing complex that breaks PCSK9 mRNA, thereby inhibiting PCSK9 synthesis (32). Therefore, it seems to be an effective drug to treat dyslipidemia and reduce the risk of MACEs (33). Like commercial PCSK9-mAbs, inclisiran is also targeting liver. In consequence, inclisiran may have no effect on HAEs in theory, and this has also been confirmed by clinical researches. ORION randomized clinical trial followed 501 subjects for half a year, whose result showed that there was no clinically meaningful change in HbA_1c_ after using inclisiran (34), indicating that there was no significant relationship between inclisiran and HAEs.

**Figure 2:**
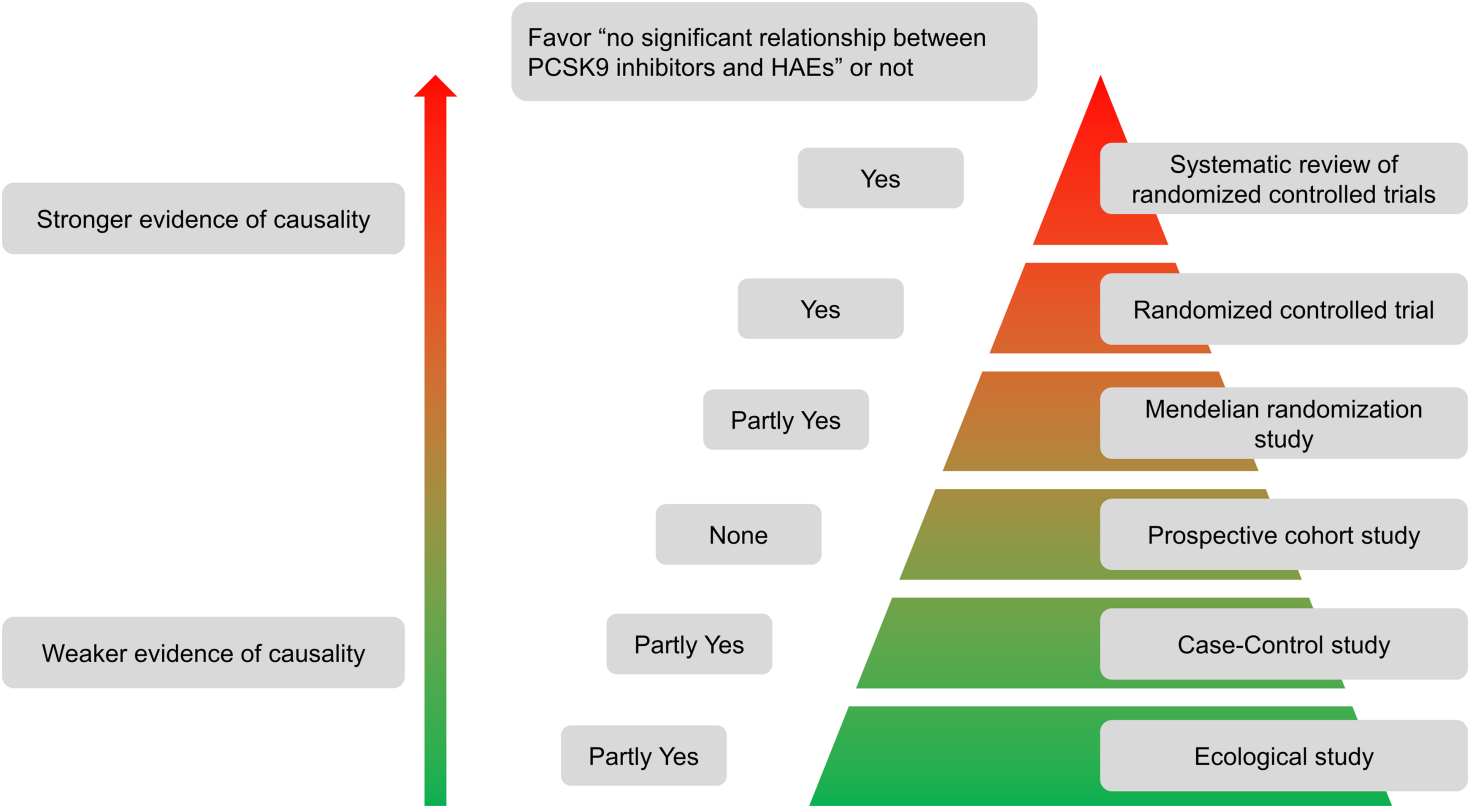
Evidence level pyramid for different study types.

According to evidence level pyramid for different study types (Figure 2), it can summarize the studies mentioned above in Table 1. In conclusion, systematic reviews of randomized controlled trials and randomized controlled trials all support that there is no significant relationship between PCSK9-mAbs and HAEs. Although the results of Mendelian randomization studies are partly contrary to that, the magnifying effect of them should not be ignored.

**Table 1:** Different study types and whether their conclusions favor “no significant relationship between PCSK9 inhibitors and HAEs”.

Study type	Author and Year	Sample size	PCSK9 inhibitor	Favor conclusion
Systematic review of randomized controlled trials	Li 2022 (30)	32 trials, 65,861 subjects	A & E	Yes
	Guedeney 2021 (29)	30 trials, 59,026 subjects	A & E	Yes
	Chen 2020 (31)	23 trials, 65,957 subjects	A & E	Yes (A reduces risk of T2DM)
	Guedeney 2019 (8)	39 trials, 66,478 subjects	A & E	Yes
	Khan 2019 (35)	12 trials, 38,933 subjects	A & E	Yes
	Monami 2019 (27)	38 trials, 89,603 subjects	A & E	Yes
	de Carvalho 2018 (28)	20 trials, 68,123 subjects	A & E	Yes (small increase in FPG and HbA_1c_)
	Cao 2018 (26)	18 trials, 26,123 subjects	A & E	Yes
	Karatasakis 2017 (36)	15 trials, 27,905 subjects	A & E	Yes
	Jones 2016 (37)	14 trials, 5,923 subjects	A	Yes
Randomized controlled trial	O'Donoghue 2022 (22)	6,635 subjects	E	Yes
	Deedwania 2021 (38)	27,342 subjects	E	Yes
	Sever 2021 (39)	27,564 subjects	E	Yes
	Schwartz 2021 (20)	13,480 subjects	A	Yes [lipoprotein(a) takes effect]
	Colhoun 2020 (40)	186 subjects	A	Yes
	Ray 2019 (41)	319 subjects	A	Yes
	Ray 2019 (18)	18,924 subjects	A	Yes
	Rosenson 2019 (42)	421 subjects	E	Yes
	Müller-Wieland 2019 (23)	458 subjects	A	Yes
	Teramoto 2019 (19)	216 subjects	A	Yes
	Lorenzatti 2019 (43)	981 subjects	E	Yes
	Chen 2019 (44)	453 subjects	E	Yes
	Ray 2018 (45)	413 subjects	A	Yes
	Ganda 2018 (46)	984 subjects	A	Yes
	Taskinen 2018 (47)	812 subjects	A	Yes
	Sabatine 2017 (21)	27,564 subjects	E	Yes
	Koren 2017 (48)	1,324 subjects	E	Yes
	Leiter 2017 (49)	517 subjects	A	Yes
	Blom 2017 (50)	901 subjects	E	Yes
	Leiter 2017 (51)	720 subjects	A	Yes
	Colhoun 2016 (17)	4,974 subjects	A	Yes
	Leiter 2019 (34)	501 subjects	I	Yes
Mendelian randomization study	Khankari 2022 (16)	–	–	Yes
	Schmidt 2017 (14)	–	–	No
	Ference 2016 (13)	–	–	No
Case-Control study	Goldman 2022 (24)	15,976 subjects	A	Yes
		71,748 subjects	E	No
Animal experiment	Da Dalt 2019 (11)	–	–	Yes
	Mbikay 2010 (10)	–	–	No

A, alirocumab; E, evolocumab; I, inclisiran.

## 3. Effects of genetic polymorphisms and variants related to PCSK9 on HAEs

Over secretion of PCSK9 resulting from PCSK9 gain-of-function (PCSK9-GOF) genetic variants may result in relative PCSK9-mAbs deficiency and lead to abnormal responses to PCSK9-mAbs. Conversely, PCSK9 loss-of-function (PCSK9-LOF) genetic variants in patients with FH may lead to unusual responses to PCSK9-mAbs because of the low relative efficacy of PCSK9 in circulation (52). So, it seems that genetic polymorphisms and variants related to PCSK9 may have effects on HAEs.

### 3.1. PCSK9-LOF genetic variants

InsLEU mutation is one of the PCSK9-LOF mutations. A genetic study in Canadian population with FH showed that, 26% of individuals in this cohort were inherited with PCSK9-InsLEU variant. There was no significant difference in lipid profiles among InsLEU-carriers when compared with non-carriers, while incidence of MACEs was significantly lower. However, higher proportion of InsLEU-carriers were found to be with prediabetes and T2DM than non-carriers. Accordingly, InsLEU-carriers had significantly higher plasma glucose levels and were more often affected with impaired FPG than non-carriers. What's more, they observed a gene dosage effect again, with homozygous or compound InsLEU-carriers having higher FPG than heterozygous InsLEU-carriers and non-carriers. Meanwhile, prevalence of impaired FPG in InsLEU gene polymorphism carriers was almost twice that in non-carriers, and proportion of homozygous or compound InsLEU-carriers with impaired FPG was higher than that of heterozygous InsLEU-carriers and non-carriers, whose trend was remarkable. In this study, after sequencing for PCSK9 exon 1 of 724 French-Canadian FH individuals carrying FH causing LDLR mutations, 188 individuals among them carried additional leucines (L10, L11), and its allelic frequency was 14% (53). Indeed, one previous study had presented that allelic frequency was 19% in a cohort composed of non-FH French-Canadian children (54). According to several studies, allelic frequency in other people ranged from 9.6% to 25% (4, 55–60).

Based on what have presented above, it can conclude that, PCSK9-LOF genetic variants are relatively common among population. In consequence, it is not necessary to test PCSK9 gene in patients before applying PCSK9-mAbs.

### 3.2. PCSK9-GOF genetic variants

It had proved that there was a link between PCSK9-GOF mutations and high plasma LDL-C levels (61), since they decreased LDLR abundance more efficiently when compared with wild-type PCSK9 (62). Based on this, PCSK9-GOF mutations could lead to FH, whose serum lipoprotein(a) level was elevated (63). A cross-sectional study including 63,320 patients in the Netherlands indicated that, incidence of T2DM in patients with FH was obviously (up to 40%) lower than that in unaffected relatives, with variability by mutation type (9).

In this way, it can reasonably believe that, different from PCSK9-LOF mutations are associated with HAEs, PCSK9-GOF mutations can decrease incidence of HAEs, although the extent of effect depends on mutation type.

### 3.3. Genes that regulate PCSK9 determine the function of PCSK9

According to the present studies, there are some genes that regulate PCSK9 and determine the function of PCSK9 (64–68).

The first one, cyclase associated protein 1 (CAP1), a new binding partner for PCSK9, has been identified and considered to be required for LDLR degradation by PCSK9. When PCSK9 catalytic domain bound LDLR, cysteine, histidine-rich domain (CHRD) of PCSK9 was found to interact with CAP1, resulted in degradation of the protein complex LDLR/PCSK9/CAP1 in lysosomes through caveolin-dependent mechanism. In this way, there are two different fates for LDLR/PCSK9 complex which depend on binding partners: degradation when dependent on caveolin pathway and recycling when dependent on clathrin pathway. What's more, prevention of PCSK9-mediated LDLR degradation could be found when used hepatocytes cultured with CAP1 siRNA and in heterozygous CAP1 KO mice. Meanwhile, PCSK9-LOF polymorphisms in human showed a defective interaction with CAP1 (64, 65). In this way, if LDLR/PCSK9 complex was degraded through caveolin pathway, the LDL-C endocytosis of hepatocytes would be weakened; on the contrary, if LDLR/PCSK9 complex was recycled through clathrin pathway, the LDL-C endocytosis of hepatocytes would be continued, and CAP1 might take an important role in this process. Based on this, the different fates for LDLR/PCSK9 complex may have effects on glycometabolism.

The other two are sterol regulatory element (SRE) binding proteins (SREBPs) and hepatocyte nuclear factor 1α (HNF1α). In a transgenic experiment, PCSK9 was observed an obvious upregulation in both male and female mice who were transformed with SREBP-1a or SREBP-2 (66, 67). This result suggests that sterol-dependent regulation of PCSK9 is mainly mediated by SREBPs. A further study identified HNF1 binding site as a critical cis-regulatory sequence in PCSK9 promoter, which was highly conserved and located between SRE and Sp1 site. It mutated the core nucleotide sequence of HNF1 site, and the data showed that disruption of HNF1 binding site reduced activity of PCSK9 promoter to only 5% of that in wild type, even lower than SRE mutation. Moreover, it also suggested that liver-enriched transcription factor HNF1*α* was the principal form of HNF1 factors that bound HNF1 sequence motifs to stimulate PCSK9 transcription (67, 68).

Therefore, it can conclude that, apart from PCSK9 itself, there are several other genes which may have effects on biological characteristics of PCSK9, and these can probably contribute to explaining why some patients developed HAEs after applying PCSK9-mAbs in some clinical researches. However, up to now, there has been no report about effects of these genes on lipid metabolism, suggesting that these gene mutations were often ignored in studies focused on lipid metabolism. In the future, sequence of these genes should be added to related researches.

## 4. Discussion

So far, animal experiments, Mendelian randomization studies, clinical researches and Meta-analyses which focused on the relationship between PCSK9-mAbs and HAEs have reached different conclusions, whose reasons can be mainly analyzed as follows.

First of all, from the study focusing on PCSK9 variants, it can conclude that insulin secretion of PCSK9^−/−^ mice is significantly lower than that of PCSK9^+/+^ mice (10), indicating that PCSK9 in systemic circulation has significant effects on function and insulin secretion of pancreatic islet β cells. In Mendelian randomization studies, the content of these studies is still about mutations of PCSK9 gene (13, 14), which is consistent with the nature of animal experiments. The results of further study based on PCSK9 KO mice also confirm it (11).

Second, circulating PCSK9 levels in PCSK9 LSKO mice decrease significantly, while PCSK9 production is normal in other organs, including pancreas (11). This effect is similar to applying PCSK9-mAbs: PCSK9-mAbs are highly targeted and can precisely inhibit PCSK9 production in liver without affecting it in other organs. This has also been confirmed by the results of many clinical researches and Meta-analyses: there is no significant relationship between PCSK9-mAbs and HAEs (17–24, 26–31). Furthermore, applying PCSK9-mAbs and ezetimibe together may help to keep glycometabolism stable, which still needs further clinical researches to confirm.

The key reason for why different kinds of experiments and researches have reached these different results is the highly targeted nature of PCSK9-mAbs, which determines that effect of PCSK9-mAbs on HAEs cannot be deduced simply and directly from conclusions about effect of PCSK9 gene variants on HAEs. At the same time, it should be noted that the reasons for this difference may not be related to duration of medication suggested in the above studies (Figure 1D).

When it comes to polymorphisms of PCSK9 gene, PCSK9-LOF variants can lead to decreased PCSK9 secretion of pancreatic islet β cells, then, impaired FPG, NOD and even T2DM. Meanwhile, PCSK9-GOF variants can lead to FH without any association with HAEs. However, since large scale cohort researches have shown that, genetic variants in PCSK9-LOF and PCSK9-GOF are relatively common (69, 70), it is not necessary to test PCSK9 gene in patients before applying PCSK9-mAbs.

At the same time, there are many other factors in the body which may have effects on biological characteristics of PCSK9, and these can probably contribute to explaining why some patients developed HAEs after applying PCSK9-mAbs in clinical researches.

There are several other published review articles on this topic. We have following advantages and differences compared with the latest review (71). First of all, although the article mentioned role of PCSK9-mAbs in pancreatic islet β cells, it ignored that currently commercial PCSK9-mAbs were targeting liver, whose effect on pancreatic islet β cells was minimal. We have paid attention to this point and believe that it is precisely because of the liver's precise targeting that there have been no adverse events of hyperglycemia. Secondly, we take effects of genetic polymorphisms of PCSK9 on HAEs into consideration, which shows polymorphisms and variants of PCSK9 can also have effects on the development of NOD. What's more, we include the newest Meta-analysis (30), which supports our conclusion “effects of PCSK9-mAbs on HAEs are not clinically significant”.

## 5. Conclusion

In conclusion, according to the results of current studies, there is no significant relationship between PCSK9-mAbs and HAEs. However, longer-term follow-up studies are still needed to confirm it. Although PCSK9 genetic polymorphisms and variants may affect the possible occurrence of HAEs, there is no need to perform relevant genetic testing before applying PCSK9-mAbs.
